# Interactions Between Donor Age and 12-Month Estimated Glomerular Filtration Rate on Allograft and Patient Outcomes After Kidney Transplantation

**DOI:** 10.3389/ti.2022.10199

**Published:** 2022-02-07

**Authors:** Wai H. Lim, Esther Ooi, Helen L. Pilmore, David W. Johnson, Stephen P. McDonald, Philip Clayton, Carmel Hawley, William R. Mulley, Ross Francis, Michael G. Collins, Bryon Jaques, Nicholas G. Larkins, Christopher E. Davies, Kate Wyburn, Steve J. Chadban, Germaine Wong

**Affiliations:** ^1^ Department of Renal Medicine, Sir Charles Gairdner Hospital, Perth, WA, Australia; ^2^ Medical School, University of Western Australia, Perth, WA, Australia; ^3^ School of Biomedical Sciences, University of Western Australia, Perth, WA, Australia; ^4^ Department of Renal Medicine, Auckland City Hospital, Auckland, New Zealand; ^5^ Department of Medicine, University of Auckland, Auckland, New Zealand; ^6^ Metro South Integrated Nephrology and Transplant Services, Princess Alexandra Hospital, Woolloongabba, QLD, Australia; ^7^ Faculty of Medicine, University of Queensland, St Lucia, QLD, Australia; ^8^ Translational Research Institute, Brisbane, QLD, Australia; ^9^ Australia and New Zealand Dialysis and Transplant Registry, Adelaide, SA, Australia; ^10^ Central and Northern Adelaide Renal and Transplantation Services, Adelaide, SA, Australia; ^11^ South Australia Health and Medical Research Institute, Adelaide, SA, Australia; ^12^ Adelaide Medical School, University of Adelaide, Adelaide, SA, Australia; ^13^ Department of Nephrology, Monash Medical Centre, Melbourne, VIC, Australia; ^14^ Department of Medicine, Monash University, Melbourne, VIC, Australia; ^15^ Western Australia Liver and Kidney Transplant Service, Sir Charles Gairdner Hospital, Perth, WA, Australia; ^16^ Department of Nephrology, Perth Children’s Hospital, Perth, WA, Australia; ^17^ Faculty of Medicine and Health, University of Sydney, Sydney, NSW, Australia; ^18^ Renal Medicine, Royal Prince Alfred Hospital, Sydney, NSW, Australia; ^19^ Centre for Kidney Research, The Children’s Hospital at Westmead, Sydney, NSW, Australia; ^20^ Department of Renal Medicine and National Pancreas Transplant Unit, Westmead Hospital, Sydney, NSW, Australia

**Keywords:** kidney transplantation, registry, allograft failure, patient and graft survival, estimated glomerular filtration rate, donor age, donor type

## Abstract

Reduced estimated glomerular filtration rate (eGFR) at 12-months after kidney transplantation is associated with increased risk of allograft loss, but it is uncertain whether donor age and types modify this relationship. Using Australia and New Zealand registry data, multivariable Cox proportional modelling was used to examine the interactive effects between donor age, types and 12-month eGFR on overall allograft loss. We included 11,095 recipients (4,423 received live-donors). Recipients with lowest 12-month eGFR (<30 ml/min/1.73 m^2^) experienced the greatest risk of allograft loss, with adjusted HR [95% CI) of 2.65 [2.38–2.95] compared to eGFR of 30–60 ml/min/1.73 m^2^; whereas the adjusted HR for highest eGFR (>60 ml/min/1.73 m^2^) was 0.67 [0.62–0.74]. The association of 12-month eGFR and allograft loss was modified by donor age (but not donor types) where a higher risk of allograft loss in recipients with lower compared with higher 12-month eGFR being most pronounced in the younger donor age groups (*p* < 0.01). Recipients with eGFR <30 ml/min/1.73 m^2^ 12-months after transplantation experienced ≥2.5-fold increased risk of overall allograft loss compared to those with eGFR of >60 ml/min/1.73 m^2^, and the magnitude of the increased risk is most marked among recipients with younger donors. Careful deliberation of other factors including donor age when considering eGFR as a surrogate for clinical endpoints is warranted.

## Graphical Abstract

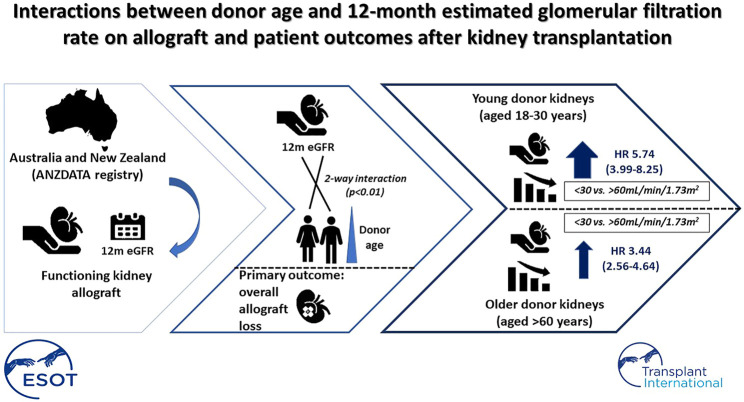

## Introduction

Reduced estimated glomerular filtration rate (eGFR) is associated with an increased risk of all-cause and cardiovascular mortality in the general population and people with chronic kidney disease (1‒4). There is an inverse relationship between post-transplant eGFR and the risks of adverse allograft outcomes in kidney transplantation, including death and death-censored allograft loss (5). Post-transplant kidney function, especially allograft function at 12-months post-transplant, is an important outcome measure and is considered one of the most critical outcomes for clinical trials in transplantation by patients and health professionals (5‒15). In a systematic review of 169 randomized controlled trials in adult kidney transplant recipients, 60% of trials utilized creatinine-derived eGFR as a study endpoint (28% and 61% as primary and secondary endpoints, respectively), emphasizing the clinical importance of allograft function as a potential surrogate measure of long-term allograft outcome ^7^.

The growing use of expanded criteria (or higher Kidney Donor Profile Index [KDPI]) donors has prompted clinicians to recognize that specific donor factors, including donor age and comorbidities, may influence short- and long-term outcomes after transplantation (16‒18). Many of these confounding factors have been adjusted for in the predictions for allograft loss and mortality. (5, 19, 20) Still, no studies have explicitly examined the potential interaction between donor factors and eGFR for these outcomes. Therefore, the aim of this study was to determine whether donor age and type modify the associations between 12-month allograft function and risk of long-term allograft and patient outcomes in a contemporary cohort of kidney transplant recipients.

## Materials and Methods

### Study Participants

All adult patients with kidney failure (aged 18 years or older) in Australia and New Zealand who had received first kidney transplants from adult living or deceased donors (aged 18 years or older) between 2000 and 2017 were included. Recipients of multiple organ allografts and those who had received prior transplants were excluded. Kidney transplant recipients with failed allografts within 12-months post-transplant and those without a recorded eGFR measurement at 12 months were excluded from the study. This study was approved by the University of Western Australia Human Research Ethics Committee (reference: 2019/RA/4/20/4584) and is reported here according to The Strengthening the Reporting of Observational Studies in Epidemiology (STROBE) guidelines (21).

### Demographics and Clinical Characteristics

Baseline characteristics included donor factors (age, donor type [living or deceased], sex, diabetes, hypertension and smoking history); recipient factors (age, sex, ethnicity, body mass index [BMI] at 12-months post-transplant, waiting time pre-transplant [in years], prevalent comorbidities [presence of diabetes, coronary artery disease, cerebrovascular disease or peripheral vascular disease pre-transplantation], smoking history and cause of kidney failure); and transplant-related factors (peak percentage panel reactive antibody [%PRA], number of human leukocyte antigen [HLA] A, B and DR mismatches, transplant era, place of transplantation [Australian states or New Zealand] and initial immunosuppressive agents).

### Exposure and Clinical Outcomes

Post-transplant kidney function, especially allograft function at 12-months post-transplant, was chosen as the exposure of interest for three reasons: 1) It is one of the most important clinical outcomes identified by both patients, caregivers and healthcare professionals (14, 15, 22); 2) Previous epidemiological studies have found a strong association between 12-month allograft function and long-term survival (5, 8, 11, 12); 3) There is established evidence to show the effect of treatments such as belatacept, on 12-month allograft function has led to improved long-term allograft survival in kidney transplant recipients (9, 23, 24). Recipients’ 12-month eGFR values were calculated using the Chronic Kidney Disease Epidemiology Collaboration (CKD-EPI) equation (25), and categorized into prior clinically defined thresholds of >60, 30 to 60 and <30 ml/min/1.73 m^2^. The primary outcome of this study was overall allograft loss (includes death-censored allograft loss and death with a functioning graft). The secondary outcomes were death-censored allograft loss, death with a functioning allograft and all-cause mortality (including death after allograft loss).

### Statistical Analysis

Data are presented as number (proportion), mean (standard deviation [SD]) and median (interquartile range [IQR]) where appropriate, with comparisons between groups examined by chi‐square test, analysis of variance (ANOVA) or Kruskal-Wallis test, respectively. The associations between 12-month eGFR, primary and secondary outcomes were examined using adjusted Cox regression models. Grouped LASSO (least absolute shrinkage and selection operator) regularized logistic regression was used for variables selection (26). The variables of importance were HLA-DR mismatches, prior smoking history, prevalent coronary artery disease, prevalent cerebrovascular disease, prevalent diabetes, primary cause of kidney failure, dialysis duration and peak %PRA in the models that considered overall and death-censored allograft loss, with the addition of recipient age and recipient smoking history in the models for death with a functioning allograft and all-cause mortality. In all Cox regression models, donor age, donor types, transplantation states and transplant era were also included as covariates.

The 12-month eGFR and donor age was considered as the two-way interaction term, and 12-month eGFR, donor age and donor types were considered as the three-way interaction term. We first tested the interaction using continuous measures of 12-month eGFR and donor age, with significant interactions (*p* < 0.01) observed for the outcome of overall allograft loss. We next constructed models evaluating the two-way interaction between categories of 12-month eGFR (according to the prior clinically defined thresholds of >60, 30 to 60 and <30 ml/min/1.73 m^2^) and donor age, with donor age thresholds informed by restricted cubic splines (5 knots; Supplementary Figure S1). There was a significant interaction (*p* < 0.1) between categories of 12-month eGFR and donor age for overall allograft loss, but not for death-censored allograft loss. However, a three-way interaction between 12-month eGFR, donor age and donor type were not observed for allograft and patient outcomes.

The estimates were expressed as adjusted hazard ratio (HR) and corresponding 95% confidence intervals (95% CI). The proportional hazard assumptions for all Cox regression models were examined graphically by Schoenfeld residuals with no evidence of departures from proportional hazards for allograft loss or mortality. A sensitivity analysis examining the associations between 12-month eGFR and outcomes were undertaken with the inclusion of other donor characteristics of diabetes, hypertension and smoking history in the Cox regression models. All analyses were undertaken using SAS (version 9.4; SAS Institute Inc., Cary, NC) and STATA (Version 15; StataCorp, College Station, TX), with *p*-values of <0.05 in two-tailed testing considered statistically significant.

## Results

**FIGURE 1:**
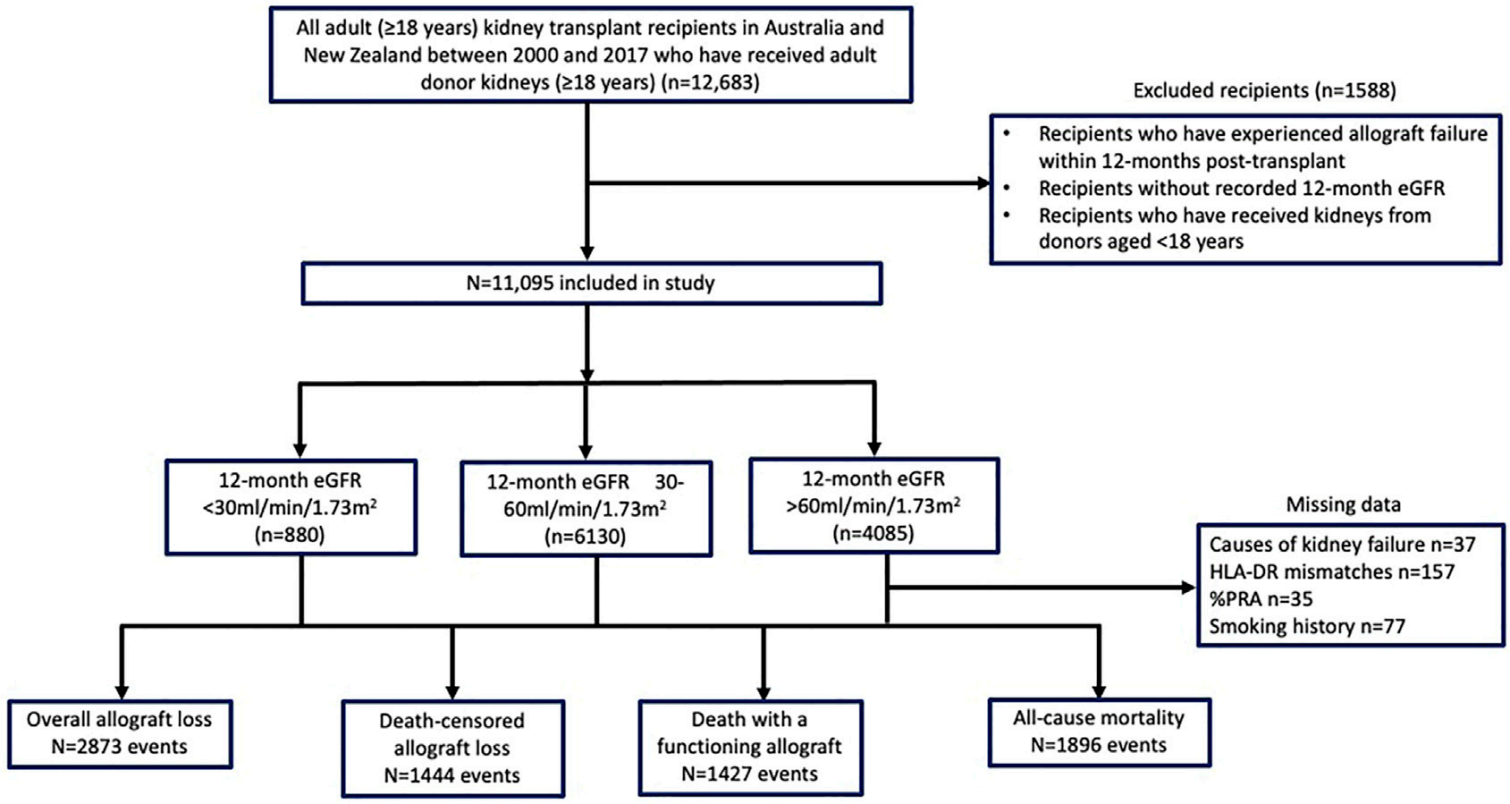
Flow diagram of the study cohort of adult kidney transplant recipients in Australia and New Zealand between 2000 and 2017.

Of the 12,683 first kidney transplants performed in 2000–2017, we excluded 1,588 recipients who lost their allografts within 12 months post-transplant or had no recorded 12-month eGFR, leaving a study cohort of 11,095 recipients (Figure 1). The mean (SD) age of the study cohort was 49 (13) years, and 37% were females. Eight hundred and eighty recipients (7.9%) had 12-month eGFR <30 ml/min/1.73 m^2^ and 4,085 (36.8%) had eGFR <30 and >60 ml/min/1.73 m^2^.

Table 1 shows the baseline characteristics of the study cohort, stratified by 12-month eGFR thresholds. Recipients with 12-month eGFR of >60 ml/min/1.73 m^2^ were younger, less likely to have prevalent vascular disease or diabetes, and had shorter mean waiting time than recipients with 12-month eGFR ≤60 ml/min/1.73 m^2^. Recipients with 12-month eGFR values of >60 ml/min/1.73 m^2^ were more likely to have received living donor kidneys and of younger donor age compared to those with 12-month eGFR of ≤60 ml/min/1.73 m^2^. The proportion of kidney transplant recipients with 12-month eGFR >60 ml/min/1.73 m^2^ increased from 30.9% between 2000 and 2004 to 38.8% between 2013 and 2017. Conversely, the proportion of recipients with 12-month eGFR <30 ml/min/1.73 m^2^ reduced from 9.4% between 2000 and 2004 to 7.7% between 2013 and 2017.

**TABLE 1:** Baseline characteristics of kidney transplant recipients transplanted between 2000 and 2017, stratified by 12-month estimated glomerular filtration rate categories.

	**eGFR categories**			*p* ***-value***
	<30 ml/min/1.73m2 (n = 880)	30–60 ml/min/1.73m2 (n = 6,130)	>60 ml/min/1.73m2 (n = 4,085)	
Recipient characteristics				
Age (year, mean ± SD)	52.4 ± 13.1	50.1 ± 12.8	46.6 ± 13.7	<0.01
Female (n, %)	351 (39.9)	2,145 (35.0)	1,561 (38.2)	<0.001
BMI (kg/m2, mean ± SD)	28.0 ± 5.9	28.2 ± 5.2	27.2 ± 5.4	<0.001
Ethnicity (n, %)				<0.001
Caucasian	668 (75.9)	4,780 (78.0)	3,000 (73.4)	
Indigenous Australian	38 (4.3)	177 (2.9)	96 (2.4)	
New Zealand Māori	19 (2.2)	171 (2.8)	85 (2.1)	
Others/not recorded	155 (17.6)	1,002 (16.3)	904 (22.1)	
Former/current smokers (n, %)	434 (49.9)	2,749 (45.1)	1,632 (40.3)	<0.001
Coronary artery disease (n, %)	117 (13.3)	642 (10.5)	325 (8.0)	<0.001
Peripheral vascular disease (n, %)	69 (7.8)	386 (6.3)	170 (4.2)	<0.001
Cerebrovascular disease (n, %)	62 (7.1)	312 (5.1)	151 (3.7)	<0.001
Diabetes (n, %)	190 (21.6)	1,031 (16.8)	637 (15.6)	<0.001
Cause of kidney failure (n, %)				0.005
Diabetes	130 (14.8)	703 (11.5)	424 (10.4)	
Glomerulonephritis	370 (42.1)	2,726 (44.6)	1782 (43.8)	
Vascular	56 (6.4)	376 (6.1)	256 (6.3)	
Cystic	131 (14.9)	1,029 (16.8)	677 (16.7)	
Analgesic Nephropathy	7 (0.8)	39 (0.6)	14 (0.3)	
Other or Unknown	185 (21.0)	1,242 (20.4)	911 (22.5)	
Waiting time (years, mean ± SD)	3.6 ± 2.9	2.8 ± 2.7	2.5 ± 2.5	<0.001
eGFR (ml/min/1.73 m2, mean ± SD)^a^	23.0 ± 5.6	46.5 ± 8.1	74.9 ± 12.6	<0.001
12-month eGFR categories (n, %)^a^				<0.001
≥90	0 (0.0)	0 (0.0)	510 (12.5)	
>60‒89	0 (0.0)	0 (0.0)	3,575 (87.5)	
45‒60	0 (0.0)	3,588 (58.5)	0 (0.0)	
30‒44	0 (0.0)	2,542 (41.5)	0 (0.0)	
15‒29	791 (89.9)	0 (0.0)	0 (0.0)	
<15	89 (10.1)	0 (0.0)	0 (0.0)	
Donor characteristics				
Age (years, mean ± SD)	57.1 ± 12.5	51.3 ± 12.4	42.4 ± 13.3	<0.001
Female (n, %)	450 (51.8)	3,126 (53.1)	1709 (43.5)	<0.001
Living donor (n, %)	201 (22.8)	2,419 (39.5)	1787 (43.7)	<0.001
Deceased DCD donor (n, %)	123 (14.0)	669 (10.9)	369 (9.0)	0.126
Donor diabetes	66 (7.5)	272 (4.4)	101 (2.5)	<0.001
Donor hypertension	312 (35.5)	1,290 (21.0)	432 (10.6)	<0.001
Donor smoking history	228 (25.9)	502 (24.5)	1,201 (29.4)	<0.001
Transplant characteristics				
HLA-ABDR mismatches (mean ± SD)	3.7 ± 1.7	3.4 ± 1.7	3.3 ± 1.7	<0.001
Ischemic time (hours, mean ± SD)	10.9 ± 6.2	8.7 ± 6.1	8.1 ± 6.0	<0.001
Peak percentage PRA (n, %)				<0.001
0–10	663 (75.5)	5,077 (83.1)	3,378 (83.0)	
11–50	140 (15.9)	667 (10.9)	463 (11.4)	
51–80	41 (4.7)	190 (3.1)	128 (3.1)	
>80	34 (3.9)	176 (2.9)	103 (2.5)	
Transplant year (n, %)				<0.001
2000–2004	221 (25.1)	1,410 (23.0)	729 (17.8)	
2005–2008	154 (17.5)	1,236 (20.2)	788 (19.3)	
2009–2012	210 (23.9)	1,441 (23.5)	1,084 (26.5)	
2013–2017	295 (33.5)	2043 (33.3)	1,484 (36.4)	
Prednisolone at 12 m (n, %)	869 (98.8)	6,055 (98.8)	4,006 (98.1)	0.012
Calcineurin-inhibitor at 12 m (n, %)				0.005
None	12 (1.4)	80 (1.3)	68 (1.7)	
Cyclosporin	161 (18.3)	1,375 (22.4)	35 (0.9)	
Tacrolimus	707 (80.3)	4,675 (76.3)	3,982 (97.4)	
Anti-metabolite at 12 m (n, %)				0.991
Non	15 (1.7)	100 (1.6)	110 (6.5)	
Azathioprine	7 (0.8)	47 (0.8)	117 (7.0)	
Mycophenolic acid	858 (97.5)	5,983 (97.6)	1,456 (86.5)	

a One-year post-transplantation.

LD, live donor; DD, deceased donor; ESKD, end-stage kidney disease, BMI, body mass index; eGFR, estimated glomerular filtration rate by Chronic Kidney Disease Epidemiology Collaboration equation; DCD, donation after circulatory death; HLA, human leukocyte antigen; PRA, panel reactive antibody; mTOR, mammalian target of rapamycin.

### Donor Age Categories and 12-Month eGFR

**FIGURE 2:**
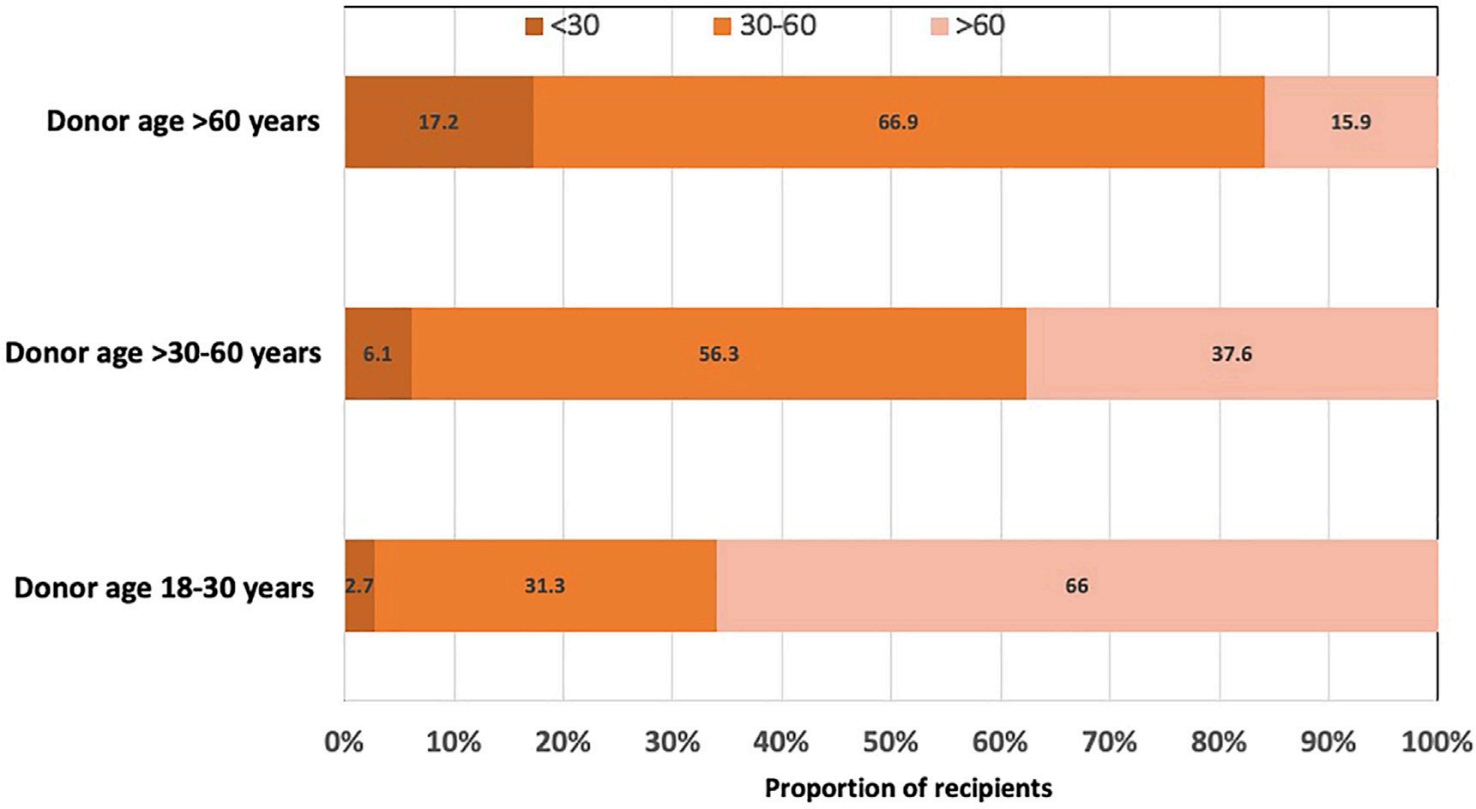
Bar graph showing the proportion of kidney transplant recipients with 12-month estimated glomerular filtration rate (eGFR) of <30, 30–60 and >60 ml/min/1.73 m^2^, stratified by donor age groups (18–30, >30–60 and >60 years).

A higher proportion of recipients who received kidneys from younger donors aged 18–30 years had 12-month eGFR >60 ml/min/1.73 m^2^ compared to recipients of donor kidneys aged >30–60 and >60 years. Conversely, approximately 17% of recipients with older donor kidneys (aged >60 years) had 12-month eGFR of <30 ml/min/1.73 m^2^ compared to 3% of recipients with younger donor kidneys (aged 18–30 years) (Figure 2 and Supplementary Table S1).

### Association Between 12-Month eGFR and Overall Allograft Loss

The estimates of the main model for overall allograft loss are shown in Table 2. Compared to recipients with 12-month eGFR of 30–60 ml/min/1.73 m^2^, recipients with the lowest 12-month eGFR (<30 ml/min/1.73 m^2^) experienced the greatest risk of overall allograft loss (adjusted HR [95% CI]: 2.65 [2.38, 2.95]); where those with the highest eGFR at 12-months experienced a lower risk of overall allograft loss (adjusted HR 0.67 [0.62–0.74]). Compared to recipients of older donor kidneys, recipients with younger donor kidneys experienced a reduced risk of overall allograft loss.

**TABLE 2:** Association between 12-month eGFR, long-term allograft and patient outcomes (main effects models).

	Overall allograft loss (adjusted HR [95% CI])	Death censored allograft loss (adjusted HR [95% CI])	Death with a functioning allograft (adjusted HR [95% CI])	All-cause mortality (adjusted HR [95% CI])
12-month eGFR (mL/min/1.73m2)				
<30	2.65 (2.38, 2.95)	3.94 (3.44, 4.53)	1.30 (1.09, 1.54)	1.78 (1.56, 2.04)
30–60	1.00	1.00	1.00	1.00
>60	0.67 (0.62, 0.74)	0.56 (0.49, 0.64)	0.82 (0.73, 0.93)	0.77 (0.69, 0.86)
Donor factors				
Live donor (ref: deceased donor)	0.92 (0.84, 1.01)	0.81 (0.72, 0.91)	0.91 (0.79, 1.05)	0.90 (0.80, 1.01)
Donor age (years)				
18–30	0.79 (0.69, 0.90)	0.69 (0.57, 0.85)	0.92 (0.77, 1.11)	0.87 (0.74, 1.02)
>30–60	0.88 (0.80, 0.97)	0.90 (0.79, 1.04)	0.90 (0.79, 1.03)	0.90 (0.80, 1.02)
>60	1.00	1.00	1.00	1.00
Recipient factors				
Recipient age (in years)	—	0.96 (0.95, 0.96)	1.07 (1.06, 1.11)	1.06 (1.05, 1.06)
Prior smoking history (ref: non-smoker)	1.30 (1.20, 1.40)	—	1.35 (1.22, 1.50)	1.37 (1.25, 1.50)
Prior coronary artery disease	1.30 (1.18, 1.44)	1.74 (1.51, 2.01)	1.03 (0.90, 1.18)	1.21 (1.08, 1.36)
Prior cerebrovascular disease	1.42 (1.25, 1.60)	1.78 (1.47, 2.15)	1.10 (0.94, 1.31)	1.20 (1.04, 1.38)
Diabetes	1.53 (1.30, 1.79)	—	1.46 (1.18, 1.80)	1.64 (1.38, 1.96)
Cause of kidney failure		—		
Glomerulonephritis	0.70 (0.60, 0.82)		0.67 (0.55, 0.82)	0.67 (0.56, 0.80)
Diabetes	0.89 (0.72, 1.12)		1.14 (0.85, 1.52)	1.07 (0.84, 1.38)
Hypertension/renovascular disease	1.00		1.00	1.00
Cystic	0.62 (0.52, 0.75)		0.79 (0.64, 0.99)	0.72 (0.59, 0.89)
Analgesic nephropathy	1.68 (1.18, 2.39)		1.53 (1.04, 2.24)	1.48 (1.03, 2.12)
Others	0.77 (0.66, 0.91)		0.85 (0.68, 1.07)	0.89 (0.73, 1.09)
Dialysis duration (in years)	1.06 (1.05, 1.08)	—	1.09 (1.07, 1.11)	1.09 (1.07, 1.11)
Transplant factors				
HLA-DR mismatches				
0	1.00	1.00	1.00	1.00
1	1.26 (1.14, 1.38)	1.59 (1.39, 1.82)	0.95 (0.84, 1.09)	1.04 (0.92, 1.17)
2	1.27 (1.14, 1.41)	1.65 (1.43, 1.92)	1.03 (0.89, 1.19)	1.15 (1.02, 1.30)
Peak PRA (%)	—		-	-
0–10		1.00		
11–50		1.24 (1.06, 1.44)		
51–80		1.31 (1.02, 1.68)		
>80		1.61 (1.26, 2.06)		
Transplant era				
2000–2004	1.00	1.00	1.00	1.00
2005–2008	1.07 (0.97, 1.18)	1.21 (1.06, 1.38)	0.90 (0.78, 1.04)	0.92 (0.82, 1.04)
2009–2012	1.06 (0.94, 1.18)	1.24 (1.06, 1.46)	0.85 (0.72, 0.99)	0.90 (0.78, 1.04)
2013–2017	1.02 (0.87, 1.19)	1.45 (1.16, 1.81)	0.68 (0.54, 0.85)	0.82 (0.67, 1.01)

Data presented as adjusted hazard ratio (HR) and 95% confidence intervals (95% CI) in the multi-variable adjusted Cox regression models, with the estimates of the covariates selected by group least absolute shrinkage and selection operator (LASSO) shown. eGFR, estimated glomerular filtration rate; PRA, panel reactive antibody; HLA-human leukocyte antigen.

### Interaction Between Donor Age, 12-Month eGFR and Overall Allograft Loss

**FIGURE 3:**
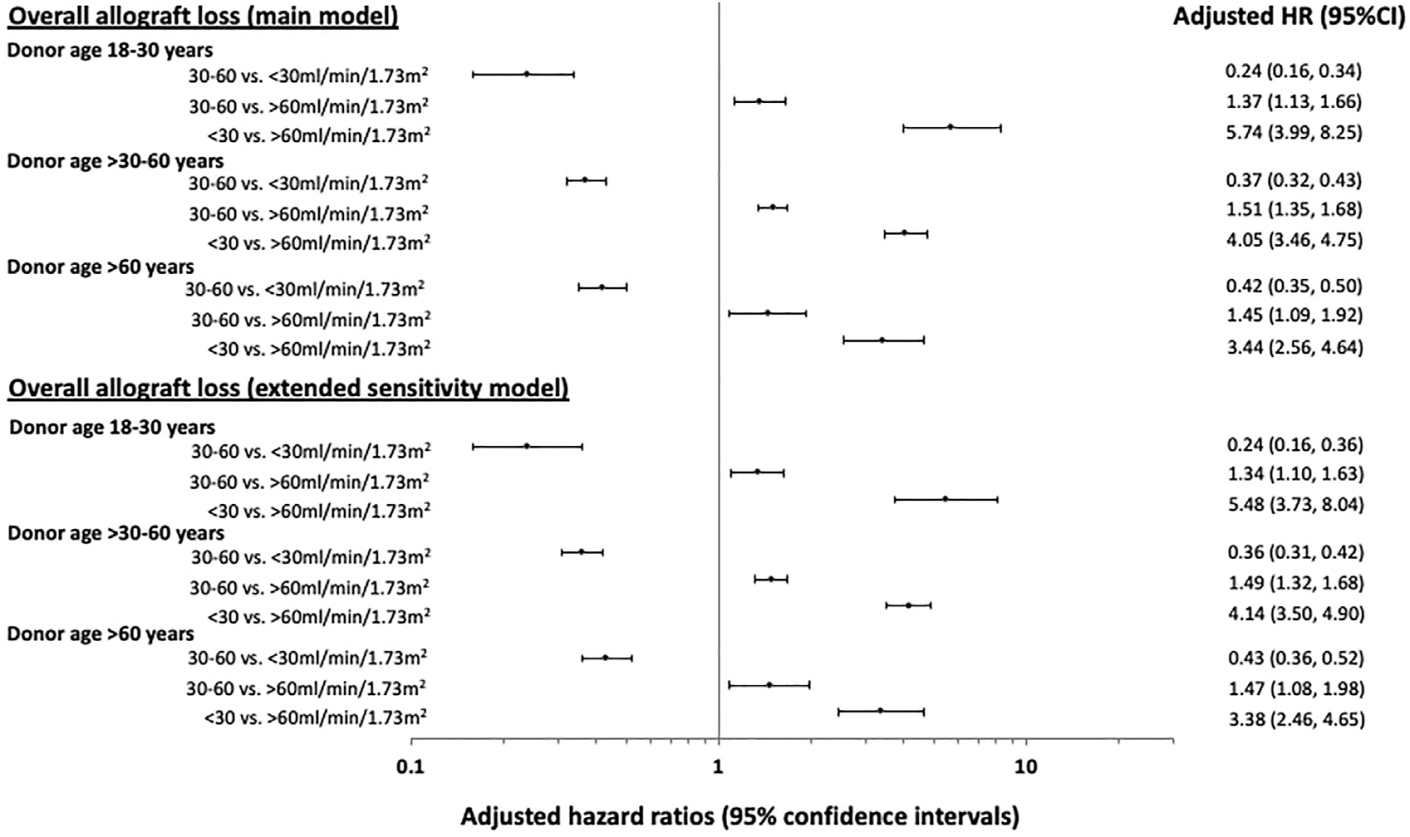
Forest plots showing the adjusted hazard ratio (HR) with 95% confidence intervals (95% CI) of the associations between 12-month estimated glomerular filtration rate (eGFR) of <30, 30–60 and >60 ml/min/1.73 m^2^ and overall allograft loss post-kidney transplantation, stratified by donor age groups of 18–30, >30–60 and >60 years. The estimates of the main and extended sensitivity models for overall allograft loss are shown.

Figure 3 shows the adjusted HRs and 95% CI for eGFR categories and overall allograft loss stratified by donor age subgroups of 18–30, >30–60 and >60 years. In recipients of kidneys from younger donors (aged 18–30 years), the adjusted HRs for overall allograft loss were highest in those with the lowest 12-month eGFR values (<30 ml/min/1.73 m^2^: HR 5.74 [95% CI 3.99, 8.25]; 30–60 ml/min/1.73 m^2^: HR 1.37 [95% CI 1.13, 1.66]; >60 ml/min/1.73 m^2^: referent). In recipients of kidneys from older donors aged >60 years, the HRs for overall allograft loss were attenuated at lower 12-month eGFR values (<30 ml/min/1.73 m^2^: HR 3.44 [95% CI 2.56, 4.64]; 30–60 ml/min/1.73 m^2^: HR 1.45 [95% CI 1.09, 1.92]; >60 ml/min/1.73 m^2^: referent) (Table 2 and Figure 3).

**FIGURE 4:**
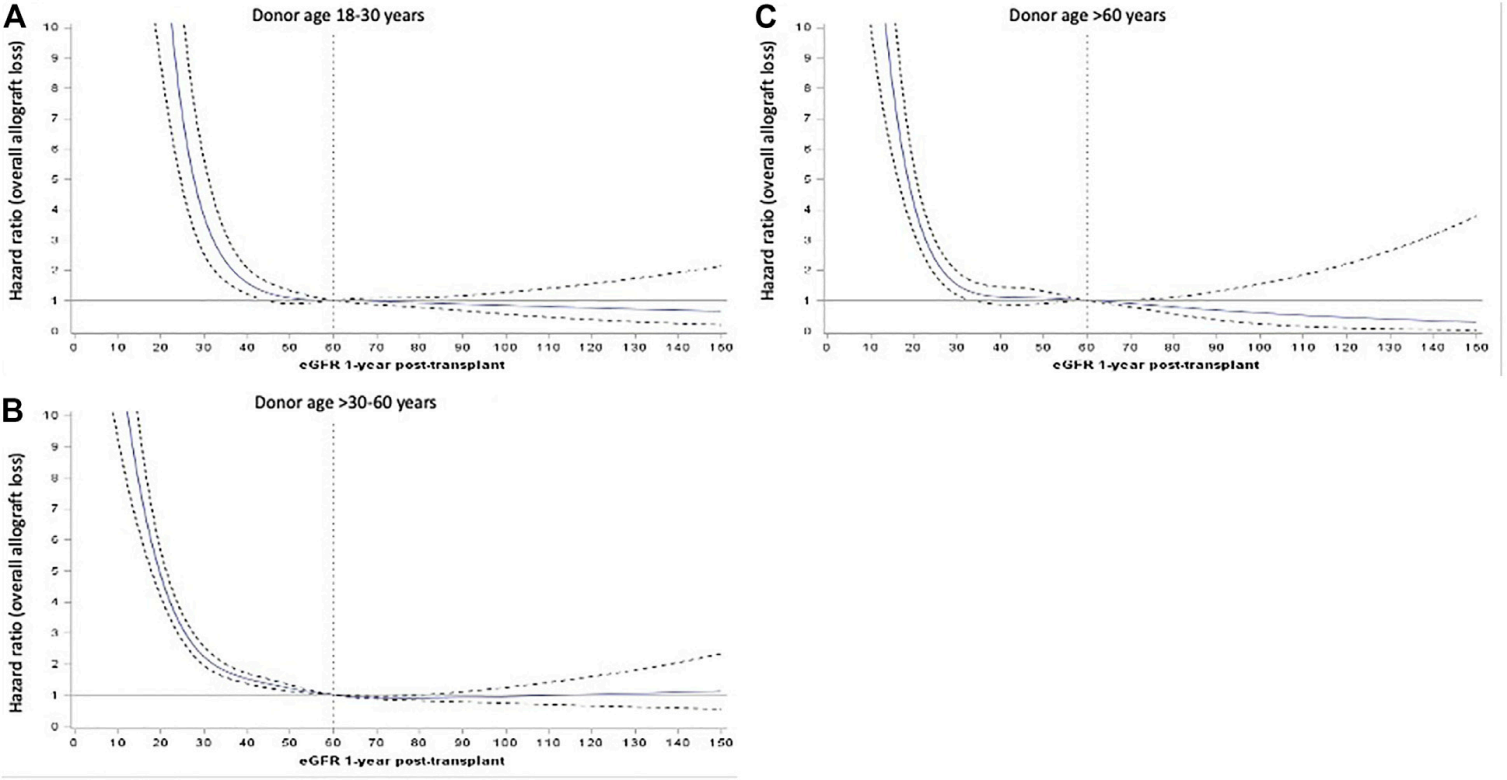
Adjusted hazard ratios (HR, represented by solid blue line) and 95% confidence intervals (95%CI, represented by dotted black lines) of the relationship between 12-month estimated glomerular filtration rate (eGFR, as continuous exposure) and hazards of overall allograft loss for donor age subgroups of 18–30 **(A)**, >30–60 **(B)** and >60 years **(C)**.

Figures 4A–C show the adjusted HR for overall allograft loss across the continuum of 12-month eGFR, stratified by donor age groups. The inflection points of the survival curves corresponding to an increased risk of overall allograft loss occurred at lower eGFR values for recipients of older donor kidneys than younger donor kidneys.

### Association Between 12-Month eGFR and Death Censored Allograft Loss, Death With a Functioning Allograft and All-Cause Mortality

The estimates of the main models (without interaction) for death censored allograft loss, death with a functioning allograft and all-cause mortality are shown in Table 2. Compared to 12-month eGFR of 30–60 ml/min/1.73 m^2^, the adjusted HR for 12-month eGFR of <30 ml/min/1.73 m^2^ was 3.94 (3.44, 4.53) for death-censored allograft loss, 1.30 (1.09, 1.54) for death with a functioning allograft and 1.78 (1.56, 2.04) for all-cause mortality. The respective HRs for 12-month eGFR of >60 ml/min/1.73 m^2^ were 0.56 (0.49, 0.64), 0.82 (0.73, 0.93) and 0.77 (0.69, 0.86). These relationships were not modified by donor age.

### Sensitivity Analysis

A greater proportion of recipients with 12-month eGFR of <30 ml/min/1.73 m^2^ received kidneys from donors with a history of diabetes or hypertension compared to recipients with higher 12-month eGFR (Table 1). In the sensitivity analysis which included these additional donor characteristics (donor diabetes, donor hypertension and donor smoking history), the two-way interaction between 12-month eGFR and donor age remained statistically significant for overall allograft loss. Figure 3 shows the adjusted HRs and 95%CI for eGFR categories and overall allograft loss according to the donor age subgroups of 18–30, >30–60 and >60 years.

## Discussion

In this contemporary cohort of kidney transplant recipients, recipients with 12-month eGFR less than 30 ml/min/1.73 m^2^ experienced at least a 2.5 fold increased risk of overall allograft loss compared to those with higher eGFR at 12 month (>60 ml/min/1.73 m^2^). This association was modified by donor age but not donor types. Recipients of younger donor kidneys with a lower 12-month eGFR value of less than 30 ml/min/1.73 m^2^ experienced up to 6-times greater risk of overall allograft loss compared to those with higher 12-month eGFR values. This association was attenuated in recipients with older donor kidneys.

Observational data shown a direct association between donor age and kidney function at 12-months and long-term allograft and patient survivals (5, 8, 20, 27‒29). but our observed interactive effects between donor age and eGFR at 12 months on allograft loss is novel. Our study findings suggest that the effects of reduced short-term allograft function at 12-month on longer term allograft outcome differs in recipients of younger and older donor kidneys, with the magnitude of the risk for overall allograft loss being higher for recipients of younger donor kidneys with lower 12-month eGFR values than those who received older donor kidneys. The inflection point for the increased risk of allograft loss occurred at a lower eGFR for older donor kidneys than younger donor kidneys. Our current findings may imply that clinical events or disease phenotypes that may have led to a reduced eGFR at 12-months for recipients with younger donor kidneys are different from recipients of older donor kidneys who had reduced eGFR at 12-months. However, these findings also suggest that donor age alone is unlikely the only contributing factor in modifying the association between 12-month eGFR and allograft outcomes. Other mechanisms or influences such as the different etiology of the allograft dysfunction (such as disease recurrence, vascular complications, cellular or antibody-mediated rejection, BK viral nephropathy), the differing susceptibility of the donor kidneys (of varying ages) to clinical insults and the presence of competing events such as death with a functioning allograft may have affected the trajectory for allograft loss for each eGFR threshold according to incremental donor age subgroups.

In a systematic review of 169 randomized controlled trials in kidney transplantation, eGFR was a primary or secondary endpoint in 60% of the trials (7). Clinical trials powered to hard clinical endpoints such as allograft survival are often not feasible in kidney transplantation. Therefore, eGFR is likely to continue to be used as a surrogate measure of allograft survival. In the two largest clinical trials ever conducted in kidney transplantation, the primary endpoint was 12-month eGFR (Efficacy Limiting Toxicity Elimination [ELITE]–Symphony study [n = 1,645]; mean [SD] donor age 45–46 [15–16] years; published 2007) or a composite of acute rejection or eGFR of <50 ml/min/1.73 m^2^ at 12-months (TRANSplant eFficacy and Safety Outcomes With an eveRolimus-based regiMen [TRANSFORM] study [n = 2037]; mean [SD] donor age of 48 [15] years, published 2018), indicating that eGFR will likely remain one of the best and practical index measures for longer-term kidney allograft outcome (11, 12). Consequently, a greater understanding of the limitations of the prognostic significance of a single timepoint eGFR is critical when considering clinical trial design and when interpreting the results of clinical trials in kidney transplantation.

Estimated GFR, however, does not necessarily provide accurate quantification of the amount and etiology of the “pathological” acute and chronic changes in the allograft biopsy, which can be influenced by multiple patient- and transplant-related factors, such as the primary cause of kidney failure, body size, age and post-transplant clinical events (e.g. disease recurrence, antibody mediated rejection) and therefore, kidney allograft biopsies are often required to guide clinical management (30). Our study suggests that donor age should be considered when interpreting the clinical applicability and prognostic significance of a single time point eGFR value such that the proportion of recipients attaining different 12-month eGFR thresholds and the association between eGFR and risk of overall allograft loss may be conditional on the effects of donor age. This finding also suggests the need for careful consideration when utilizing a single time point eGFR value as a surrogate measure for overall allograft loss in kidney transplant trials.

There are several strengths and limitations in this study. The prospective nature of a contemporary cohort of kidney transplant recipients and the near completeness of the available data suggest that ascertainment biases of the exposure and outcome measures were minimized and that the study findings reflect current clinical practice. Indication bias remained a possibility because there may have been systematic differences in how clinicians manage kidney transplant recipients with differing eGFR values at 12-months post-transplant. However, the direction of this bias is likely towards the null hypothesis because people with lower eGFR may receive closer monitoring or changes to the management approach due to the lower eGFR. Even though there were multiple confounding factors adjusted for in the analyses, there are likely to be several unmeasured and residual confounders. These include the overall exposure and utilization of immunosuppression (according to clinical risk), the impact of various adverse clinical events/hospitalizations occurring during the time course of the follow-up period, lack of availability of biopsy data and changing nature of immunological risk (such as evidence of transplant glomerulopathy, presence of interstitial fibrosis/tubular atrophy, development of *de novo* donor-specific anti-HLA antibody), presence of and severity of proteinuria and the development (and severity) of *de novo* comorbid conditions such as post-transplant diabetes and hypertension that may have influenced allograft function and allograft survival post-transplant; which were not adequately collected by the ANZDATA registry but may potentially have modified our study findings. It was determined *a priori* that change in eGFR would not be considered in this study given that the majority of landmark clinical studies had utilized a single time point eGFR measurement as the primary or secondary endpoint. However, our other work has shown that change in eGFR is a valuable predictor of long-term outcomes (19). Misclassification bias of actual allograft function may have occurred, however, measured GFR was impractical and costly in the real-world setting. In addition, given the small number of kidney transplant recipients of younger deceased donor kidneys that achieved 12-month eGFR values of <30 ml/min/1.73 m^2^, there is likely considerable uncertainty in the estimates to provide an accurate assessment of the true difference between eGFR and allograft outcomes for this group.

In conclusions, our study shows that the association between 12-month eGFR and allograft outcome is modified by donor age. Even though the relationship between eGFR and allograft outcome is similar among different donor age subgroups, an identical single timepoint eGFR as a prognostic indicator of allograft survival and the attainment of a range of eGFR thresholds varies according to these subgroups.

## Capsule Sentence Summary

Reduced estimated glomerular filtration rate (eGFR) 12-month post-transplant is associated with adverse long-term allograft outcomes but whether donor factors such as age, modify this association in unknown. Using data from the Australia and New Zealand Dialysis and Transplant (ANZDATA) registry, we have shown that the relationship between 12-month eGFR and allograft loss was modified by donor age. Even though the trend and nature of the relationships between 12-month eGFR and allograft loss were similar, the magnitudes of the risk were dissimilar among donor age subgroups.

## Data Availability

The data analyzed in this study is subject to the following licenses/restrictions: Use of deidentified data can be requested from ANZDATA registry. Requests to access these datasets should be directed to requests@anzdata.org.au.
