# Supplementary material for: Interactions Between Donor Age and 12-Month Estimated Glomerular Filtration Rate on Allograft and Patient Outcomes After Kidney Transplantation
Source: Transpl Int. 2022 Feb 7;35:10199. doi: 10.3389/ti.2022.10199 (PMC8842263; doi:10.3389/ti.2022.10199)

Supplementary Table 1. Proportion of kidney transplant recipients within each 12-month estimated glomerular filtration rate (eGFR) category, stratified by donor age subgroups of 18-30, >30-60 and >60 years.

	Donor age groups		
	18-30 years	>30-60 years	>60 years
**eGFR categories**			
<30mL/min/1.73m^2	38 (2.7%)	455 (6.1%)	387 (17.3%)
30-60mL/min/1.73m^2	442 (31.3%)	4189 (56.3%)	1499 (66.8%)
>60mL/min/1.73m^2	930 (66.0%)	2799 (37.6%)	356 (15.9%)
**Total**	1410 (100.0%)	7443 (100.0%)	2242 (100.0%)

Supplementary Figure 1. Restricted cubic spline showing the relationship between donor age and overall allograft loss.

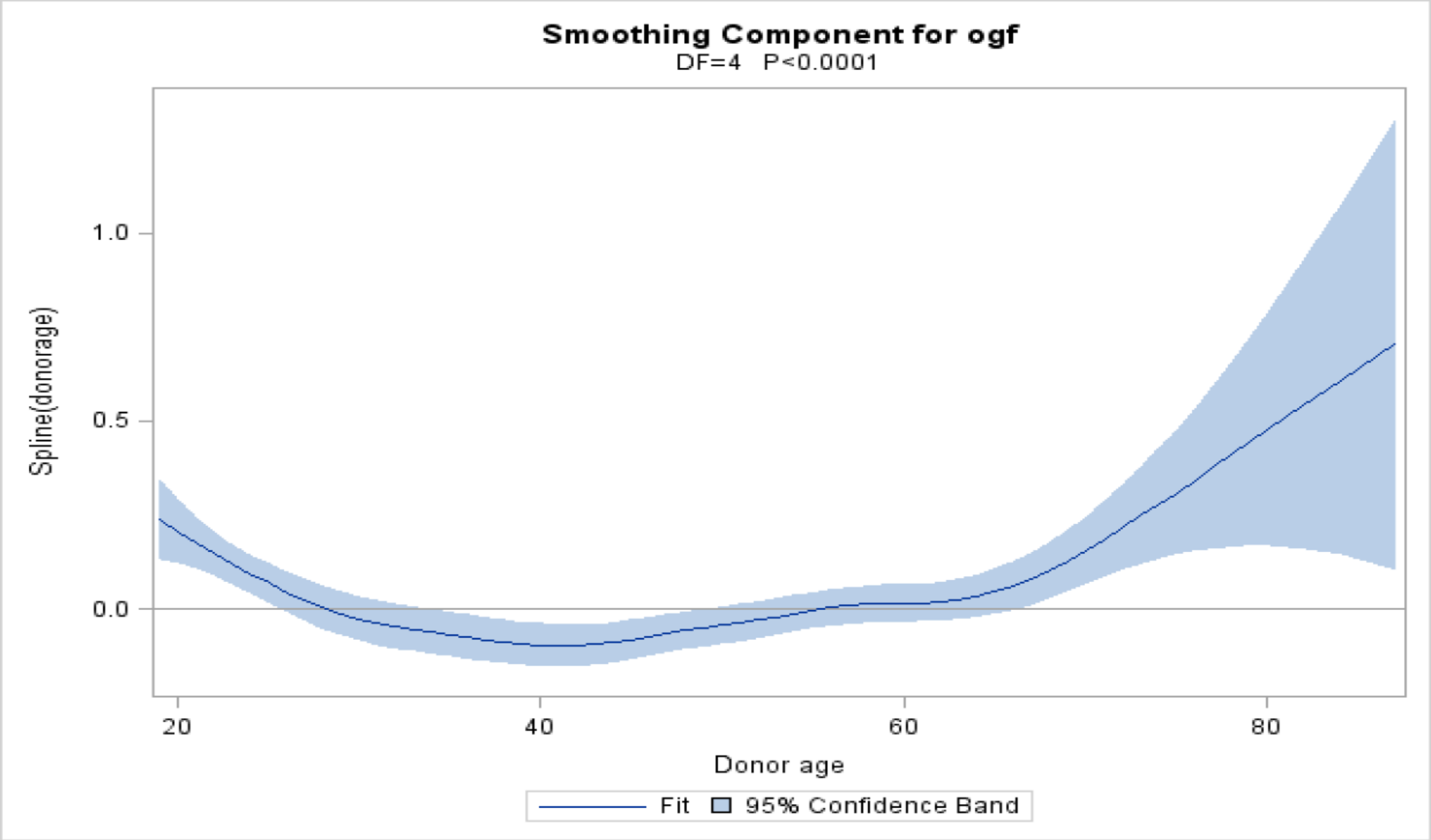

Supplement: Supplementary file 1 [file DataSheet1.PDF]
